# Parents’ experiences of mathematics learning at home during the COVID-19 pandemic: a typology of parental engagement in mathematics education

**DOI:** 10.1007/s10649-023-10224-1

**Published:** 2023-04-03

**Authors:** Steve Murphy, Lena Danaia, Jacquie Tinkler, Fiona Collins

**Affiliations:** 1 School of Education, La Trobe University, Wodonga, Victoria Australia; 2 School of Education, Charles Sturt University, Bathurst, New South Wales Australia; 3 School of Education, Charles Sturt University, Wagga Wagga, New South Wales Australia

**Keywords:** Mathematics learning, COVID pandemic, Parental engagement, Home learning, Emotional capital

## Abstract

The COVID pandemic disrupted the schooling of students worldwide resulting in many having had a period of at-home learning. Many parents found themselves assuming responsibility for supporting their children’s at-home learning. Parents often find it difficult to support their children’s mathematics learning compared with other curriculum areas. There has been limited research exploring parental engagement in mathematics education generally, and little into parental engagement in mathematics education during the COVID pandemic. This paper examines how parents supported their child’s mathematics education during the school closures and identifies the factors that impacted this engagement. The Ecologies of Parental Engagement (EPE) model was used to help describe the engagement of different parents in mathematics education during the school closures and to examine the way the home space and available capital shaped parental engagement. Eight parents were selected from a larger Australian study that explored the impact of the pandemic-induced period of at-home schooling on primary school mathematics and science. One-on-one narrative interviews were conducted online with participants. Analysis identified three categories of parental engagement: monitors, facilitators, and enhancers. Parents in each category responded to their role in at-home learning differently, and accessed and activated different capital to support their child’s at-home learning in mathematics during the pandemic. Results highlight the value of emotional capital, as well as knowledge of mathematics and mathematics education, with implications for schools hoping to engage parents in mathematics learning. The study offers a typology to be explored in future research concerning parental engagement in mathematics education.

## Introduction

The COVID-19 pandemic caused jurisdictions worldwide to close their schools, disrupting the schooling of approximately 1.6 billion children (OECD, 2020). Internationally, these closures interrupted children’s learning and exacerbated equity issues in education (Doyle, 2020; Gore et al., 2021; UNESCO, 2020). Some authors anticipated that mathematics education would be particularly impacted by the COVID-19 school closures, extrapolating from the negative impact on mathematics achievement of prolonged absences from school associated with the summer holidays (Reimers & Schleicher, 2020). Early indicators suggest these predictions may have come to bear, with lower progress in mathematics experienced by students from schools serving low socioeconomic communities in the USA (Opportunity Insights, 2021) and in Australia (Gore et al., 2021).

Worldwide, children’s parents and carers found themselves tasked with responsibility for supporting “at-home” learning. Mathematics education is likely to have been particularly impacted by this transition as parents generally find it more difficult to support their children in mathematics compared to other learning areas (Jay et al., 2018). Furthermore, the way parents engage in their children’s mathematics learning determines the impact of this engagement. When parents express interest in children’s mathematics tasks and show confidence in their children’s mathematics abilities, there is a positive impact on learning (Rodríguez et al., 2017). However, where parents focus upon monitoring of schoolwork, or where parents offer support using strategies different from those at school, parental engagement in mathematics learning can have negative impacts (Patall et al., 2008).

While the relationship between the type of parental engagement and children’s mathematics learning is explored in the literature, research into factors influencing this engagement is more limited. It is generally accepted that employment conditions, care-giving responsibilities, and broader life context all impact parental engagement in schooling generally (Posey-Maddox & Haley-Lock, 2020); however, factors contributing to parental engagement in mathematics specifically are less well understood. The COVID-induced school closures, where parents largely assumed responsibility for supervising and supporting their children’s formal mathematics learning, have created an opportunity to deepen our understanding of parental engagement in their children’s mathematics education. This paper reports on research that exploits this opportunity. Through the analysis of in-depth, narrative interviews with eight Australian parents about their experiences of at-home mathematics education during the school closures, this paper explores the following research questions:

1. How did parents engage in mathematics education during the COVID-19 school closures?
2. What factors impacted this engagement?

The findings have implications for how parental engagement in children’s mathematics education can be supported both during school closures and during the normal operation of schools.

## Background

In this paper, we use the term “parent” to denote the people, including carers and other family members, who adopted a primary role and responsibility for the at-home support of children’s formal schooling. Furthermore, there are a range of terms used to describe the ways parents contribute to the formal education of their children, including “parental involvement”, “parent-school partnership”, and “parent engagement” (Woodrow et al., 2016). In this paper, we use “parental engagement” as a catch-all term for the actions of parents relative to their children’s schooling.

### Parental engagement in mathematics education

The limited research into parental engagement in mathematics education suggests varied impacts dependent on the type of engagement. Parents communicating interest in mathematics work and progress, and confidence in their children’s ability has a positive impact on student achievement (Rodríguez et al., 2017). Home support and expectations have been found to be associated with a reduction in children’s mathematical anxiety, which in turn positively impacts student performance in higher level mathematics tasks such as word problems and pre-algebraic reasoning (Vukovic et al., 2013). Parental engagement in mathematics can also impact negatively. Parents merely monitoring mathematics homework can negatively influence student achievement (Patall et al., 2008). However, parents becoming overinvolved by directly assisting with mathematics tasks can also negatively impact mathematics achievement (Rodríguez et al., 2017), particularly when assistance conflicts with the instructional techniques used at school (Patall et al., 2008). Some research suggests that when parents supporting mathematics homework offer instruction that is different from that provided at school or beyond the child’s capability, it risks the child experiencing emotional trauma (Lange & Meaney, 2011).

### Factors impacting parent engagement with their children’s mathematics education

A complex range of factors are known to influence parental engagement in schooling generally, including employment conditions, care-giving responsibilities, and broader life context (Posey-Maddox & Haley-Lock, 2020). Parents from low-SES backgrounds report feeling unsure and ill-prepared to take a role in their children’s formal schooling (Woodrow et al., 2016). Some parents also report emotional difficulties and the fear of being judged associated with engaging in their children’s schooling (Woodrow et al., 2016). There is some evidence that these general factors impact parental engagement in their children’s mathematics education. For example, Jay et al. (2018) found that access to books, online materials, private tutors, and family members impacted parents’ engagement with mathematics learning.

There is some limited research indicating parents’ mathematical knowledge and confidence impacts engagement in their children’s mathematics learning. Parents with stronger backgrounds in mathematics are generally better at conveying mathematical ideas to their children and scaffolding their children’s mathematics learning (Hyde et al., 2006). Parents who are mathematicians are more likely than non-mathematicians to provide their children with a breadth of mathematical experiences and to involve themselves in their children’s mathematics homework (Antolin Drešar & Lipovec, 2017). Parents’ confidence in mathematics shapes their approach to supporting their children in mathematics (Muir, 2012). Parents who feel confident in mathematics are more likely to become involved in their children’s mathematics education than those who do not feel confident (Antolin Drešar & Lipovec, 2017), and parents with higher confidence in mathematics tend to provide more effective support to their children (Hyde et al., 2006). Conversely, parents with poor experiences of, or a lack of confidence in, mathematics can be reluctant to engage with their children’s mathematics education (Anthony & Walshaw, 2009; Jay et al., 2017).

There is also some evidence that parents’ familiarity with contemporary mathematics education practices impacts their engagement in mathematics education. Some parents experience frustration with unfamiliar mathematical problem-solving methods that their children are taught at school, leading to reluctance to engage with their children’s mathematics learning (Jay et al., 2018). Differences between parents’ experiences of mathematics education and the mathematics education practices at their children’s schools are widely viewed as impacting negatively on parental engagement (Anthony & Walshaw, 2009; Antolin Drešar & Lipovec, 2017).

## Conceptual framework

To explore parental engagement in mathematics schooling during the school closures, we use a conceptual lens informed by the Ecologies of Parental Engagement (EPE) model (Calabrese Barton et al., 2004). EPE describes the complexities shaping parental engagement in schooling, considering the impact of both space and capital. Space is made of underlying structures and resources organised in a way recognisable to the individuals in the space. Individuals gather in a space for a particular purpose and assume roles and interact in ways constrained by norms and expectations. They share the use of tools and the production of artefacts typical of the space. Before the COVID school closures, spaces for academic mathematics learning were typically classrooms where teachers and students gathered to teach and learn mathematics, following a mathematics curriculum, employing a set of classroom mathematics resources, and producing models, posters, worksheets, and tests. Parents had only a very limited role in the mathematics learning space, largely limited to some involvement with homework and receiving student reports, and very limited access to the associated physical space, resources, and understanding of norms and expectations.

The actions of individuals in a space are further influenced by the capital they can access and activate in that space (Calabrese Barton et al., 2004). The EPE model describes capital as “the material resources, social networks, beliefs, and personal life orientations on which people draw to direct their actions” (Pérez Carreón et al., 2005, p. 468). For example, capital typically activated in an academic mathematics learning space includes resources such as digital technology and materials to model mathematics problems; knowledge of mathematics, mathematics curriculum, and contemporary mathematics pedagogies; connections with others in the classroom, the school, and beyond; and personal experiences and beliefs about mathematics. Given this list, it could be argued that parents generally have limited access to the capital generally activated in academic mathematics learning spaces. However, parents have different forms of capital that can be mobilised to gain influence and control in a particular space (Calabrese Barton et al., 2004). The literature associated with parental engagement in mathematics education discussed above provides examples of parents activating a range of capital to engage in their children’s mathematics education. Some parents activate material capital (Calabrese Barton et al., 2004) to provide physical resources or hire tutors with additional expertise. Parents draw on their social capital (Robison et al., 2002), including their connections with family, friends, and school staff, to facilitate their engagement with their children’s mathematics education. It seems that emotional capital (Reay, 2004) is drawn upon as parents manage their own emotional responses to mathematics education while endeavouring to be supportive, patient, and empowering for their children. Finally, some parents have access to useful cultural capital (Reay, 2004) including knowledge and skills in mathematics and/or mathematics education.

Viewed through the EPE lens, the COVID-induced school closures were a significant disruption of the space and capital associated with parental engagement in academic mathematics learning. The home became the space for mathematics learning, a space occupied by different individuals with different roles and governed by different rules to the classroom. Parents who previously had limited to no role in academic mathematics learning were made central to this process. Furthermore, prior to the school closures, academic mathematics learning required relatively limited activation of capital by parents, whereas during the closures, parents were required to draw significantly on their capital resources.

This disruption provided a unique opportunity to examine parental engagement in academic mathematics learning. The rules and roles of the classroom space that traditionally shape (and restrict) parental engagement were broken down, and parents were required to mobilise significant capital to engage in their children’s mathematics learning. This study capitalises on this disruption to examine parental engagement with their children’s mathematics learning, and the ways space and capital shaped this engagement.

## Method

The present study is part of a larger research project exploring the impact of the pandemic-induced period of at-home schooling on primary school mathematics and science in Australia’s three most populous jurisdictions: New South Wales (NSW), Queensland, and Victoria. All jurisdictions closed their schools to all but vulnerable children and children of essential workers in late-March 2020 (Gore et al., 2021; Wright, 2021; Zillman, 2020). Schools remained closed for approximately two months, a period that included a regular two-week school holiday. The reopening of schools varied between the jurisdictions with all NSW students returning to school on the 25 May (National Centre for Immunisation Research & Surveillance, 2020), Queensland kindergarten and Grade 1 students returning on the 11 May, and the remainder of primary school students returning on the 25 May (Zillman, 2020), and Victorian kindergarten, grade 1, and grade 2 students returning on the 26 May 2020, and grades 3 to 6 returning two weeks later on the 9 June 2020 (Wright, 2021). Victoria experienced a subsequent COVID wave in 2020 resulting in schools in metropolitan Melbourne closing again on the 20 July 2020, with the rest of the state following suit on the 5 August 2020. Given this, the Victorian participants in this study were supervising at-home schooling when they were interviewed, whereas the NSW and Queensland participants’ children were back at school.

Thirty-two primary school teachers and 43 parents of primary school students shared their experiences through two separate Facebook groups, and through a series of one-on-one narrative interviews. The Facebook groups were established using digital snowballing sampling (Bhutta, 2012), where researchers used professional networks and other Facebook groups to recruit initial participants, and these participants were encouraged to invite others to participate in the research.

### Participants

A purposive sample of eight parents were recruited to be interviewed. This sample was built to include participants from NSW, Queensland, and Victoria. Furthermore, the parents in this sample made comments about their experiences of at-home mathematics to the Facebook group that were representative of the diversity of comments made by parents within the larger Facebook group. Beitin (2012) suggests a sample of interview participants of this size is sufficient for saturation. It is worth noting that all participants were mothers. These parents are listed by their pseudonym in Table 1, along with the number and grade level of their primary school aged children.

**Table 1:** Participants and the number and grade-level of their primary school-aged children, grouped by location

Participant pseudonym	Location	Number and grade level of primary school-aged children
Anna	New South Wales	2 children, grades 4 and 6
Sarah	New South Wales	1 child, grade 5
Denise	Queensland	2 children, grades 1 and 5
Courtney	Victoria	2 children, grades 1 and 4
Lois	Victoria	1 child, grade 6
May	Victoria	3 children, grades K, 3 and 4
Theresa	Victoria	2 children, grades 2 and 5
Penelope	Victoria	3 children, grades 1, 4, and 6

*K*, kindergarten; first grade of formal schooling in Australia

### Data collection

Participants were interviewed using an open narrative inquiry approach (Kim, 2016). This approach privileges the experiences and perceptions of the participant. Participants were then given the option to tell their story of at-home mathematics learning in their own way, or the interviewer could ask them questions. Two participants, Anna and Lois, opted to begin their story without questions, while the others requested that the interviewer ask questions. Questions provided only a chronological structure for participant’s narratives. They asked about the experiences of mathematics education of the participants and their children before school closures, during school closures, and after school closures. This approach supported the sharing of the participant’s perspective without risking leading their responses (Morse, 2012).

Interviews took place between August and September 2020, after school had returned following the first wave of shutdowns in Australia, but during Victoria’s second wave of school closures. All interviews took between 30 minutes and one hour, were conducted via video or phone call, and were audio recorded, except for Courtney, who opted to share her experiences via a series of three emails to the interviewer over a period of two weeks. The interviews were transcribed, and interview transcriptions were used for analysis.

### Data analysis

In contrast to thematic analysis, where interview data is fragmented into categories to provide evidence of common themes (Clarke & Braun, 2017), the approach to narrative analysis employed here takes the sometimes disjointed and non-linear interview data to create a cohesive narrative of each participant’s experience (Martinie et al., 2016). Through iterative engagement with the transcript of each participant’s interview and recording, a researcher wrote a brief narrative (between 600 and 1000 words), or synopsis, of each participant’s experiences of at-home mathematics learning. These synopses were independently compared to the interview data by two other researchers, and amended where necessary, until there was consensus that each was a faithful account of the participant’s experience (Kim, 2016) of at-home mathematics learning. Each synopsis followed a consistent format. First, interview data were used to create a description of both the participant’s and the participant’s children’s experiences of, and thoughts and feelings about, mathematics and mathematics education prior to the pandemic. This coverage allowed identification of pre-existing capital available, particularly as it relates to mathematics education. Next, an overview of participant and their children’s experiences during the school closures was developed, noting actions they took and reflections they made. This section captured details of parental engagement in mathematics learning, and the impact of space and capital available and activated. Finally, a brief reflection on the return to school once schools re-opened was included. Adopting such a consistent format allowed each to act as a comparable synoptic unit that preserved the relationship between key data for analysis (Hopwood, 2018).

Two researchers independently examined these synoptic units to seek common patterns of parental engagement demonstrated by each participant in their children’s mathematics education. That is, participants were grouped according to the common actions they described taking regarding their children’s mathematics learning during at-home learning. The researchers then held a consensus meeting to agree upon the categorisation of the engagement of participants. It was determined that participants demonstrated characteristic engagement with their children’s mathematics learning that fell into one of three categories. Some parents primarily monitored or enforced the completion of set mathematics work and were categorised as “monitors”. Other parents worked alongside their children as they did their mathematics work, coaching and attempting to motivate their children, and were categorised as “facilitators”. Finally, some parents critiqued the work set, and modified or supplemented work accordingly, and were categorised as “enhancers”. The parents grouped into each category, along with comments illustrating why each parent was categorised as they were, are shown in Table 2.

**Table 2:** Participants categorised by parental engagement in their children’s mathematics education during the school closures

Engagement category	Description of typical action	Participant pseudonym	Comments illustrating categorisation
Monitors	Monitoring at-home mathematics tasks without becoming significantly involved in the tasks	Denise	“…they hated doing the school work for me. … the oldest one, I had to get her teacher to give her a call a couple times and tell her to pull her head in a little bit.”
		Sarah	“…he’d just get up and do it and do the bare minimum what he had to do and he’s done for the day… I think he got a … bit careless because I would mark it—you could mark it out of the back of the textbook—and some of it was a bit wrong because he just rushed it…”
Facilitators	Supporting at-home mathematics tasks by working alongside their children on the tasks when required	Lois	“…we chose three of them by trying to choose a couple of easy ones for him to do on Tuesday and Thursday when I'm at work … then something like that, you know needs a bit of guidance or enthusiasm he’ll do on the day that I’m at home …”
		May	“I get online each day with my son who's with the preps…help my son stay connected”
		Theresa	“…it took a lot of my time … just making him understand what the activity meant… I have told him, look, if you’ve got questions, you come and ask me …”
Enhancers	Assessing the suitability of at-home mathematics tasks and modifying/supplementing these tasks when required	Anna	“We did… some stuff together at home that … wasn’t stuff that the teacher had set… he needed that modelling to be able to understand what to do before completing the worksheet…”
		Courtney	“…he flew through all the Math activity books available at Kmart, Big W and the like as well as numerous maths sheets made up by parents and grandparents and printed off the internet.”
		Penelope	“I could look at the kids and think you have not got any lights on about this topic. And so then we try and make it a bit more applied.”

The Ecologies of Parental Engagement (Calabrese Barton et al., 2004) posits that engagement is the mediation of space and capital. Using the EPE lens, in order to examine how the engagement of participants had been shaped, the researchers applied thematic analysis (Clarke & Braun, 2017) to the data to examine the impact of space and capital. This analysis identified some common impacts of space and capital across the engagement categories, as well as impacts unique to each category.

## Results

The following results present an analysis of each engagement category identified—monitors, facilitators, and enhancers—through the lens of EPE. The results describe the engagement of participants in each category, the impact of space on this engagement, and the capital activated by these participants.

### Monitors

Sarah and Denise were categorised as monitors. Sarah had one primary school aged child, a son in grade six, and three much older children no longer attending school. Denise had two daughters, both in primary school, in grade 1 and grade 5.

#### Engagement

The monitors primarily described their role in at-home mathematics learning as ensuring the school-set tasks were completed. Sarah’s role seemed to be mostly checking that her son had attempted the set tasks. She said,

I would mark it… out of the back of the textbook and some of it was a bit wrong because he just rushed it or he wouldn't ask for help. So but he didn't care, so long as I had something to upload to the teacher.

Sarah said that her son “really enjoys maths” and she did not report having to support her son with difficulties with understanding, or completing, mathematics tasks. Denise seemed to supervise her daughters more actively than Sarah; however, her focus was also on completion of tasks. She said, “we just sort of worked through lunch and morning tea and tried to get it all done so we could just do nothing”. Unlike Sarah, Denise found supporting at-home learning “pretty stressful” and “frustrating”. She said, “getting them to sit and do it with me was hard”.

The monitors did not believe that their engagement in their children’s at-home mathematics learning had contributed to learning gains, but they believed it had not resulted in a learning loss. Sarah said that during shutdown, her son became “a bit lazy” and “a bit careless”; however, she said that at-home learning “didn’t get him back any in any way”. Denise similarly noted no progress but no learning loss in her daughters’ mathematics learning, saying, “I don’t think they were getting anything out of what I was teaching them”, while also asserting “mine came out of it pretty good”.

#### Space

Both monitors used the home space to incentivise the completion of mathematics work by allowing their children free time once tasks were completed. Sarah said her son would “just get up and do it and do the bare minimum what he had to do and he was done for the day”. Similarly, Denise said that once schoolwork was completed, her daughters “just did whatever they wanted for the rest of the day”. Beyond this, they continued to take the role of deferring to the teacher to direct all learning activity. Sarah described her son addressing the tasks as they were posted by the teacher online daily. Denise worked to ensure all set tasks were completed despite noting that the work was “really boring” and that her daughters have “already done it, or they already knew it”.

#### Capital

The monitors had relatively limited capital associated with mathematics or education to use to support their children. Sarah, in describing her experience of mathematics at school, said, “I never really excelled greatly at it… it’s not one of my most favourite subjects”. She counted herself fortunate that her son was good at mathematics. She used the answer schemes provided by the school to assess her son’s work; however, she did not intervene in anyway if work was not up to standard. She provided some explanation for this, saying, “I kind of believe homework is a bit of a waste of time, at that age anyway. So, I never really push it”. Similarly, Denise revealed that she had limited mathematics or education-associated capital to activate to support at-home mathematics learning. She did not view herself as good at mathematics, explaining that she had left school in grade 11, and saying that she was relieved she did not have to support her children with high school mathematics. She also struggled to engage with the school’s mathematics education practices that involved digital resources. Instead of using the resources made available online, Denise requested printouts, saying, “they gave me some website to go to, but that was just overwhelming, so they printed it all out for us”.

While the monitors had limited mathematics or education-related capital, they did have, and activated, other capital to engage in at-home mathematics learning. Sarah worked two days per week, so was able to supervise at-home learning on other days. Denise actually left work, “to keep the kids home because my husband made me”. They also both involved family members in at-home mathematics learning. An older sibling supervised at-home learning two days a week while Sarah worked. Denise involved her partner in the more difficult work that her eldest daughter was set, saying, “I generally left that to Dad because she listens to Dad more than me”. Denise also identified some deficits in capital that impacted at-home mathematics learning, primarily associated with her own digital literacy, “I couldn’t be bothered juggling with two computers. So we got the paper version of everything”.

### Facilitators

Lois, Theresa, and May were categorised as facilitators. Lois had a son in grade 6. Theresa had two sons, one in grade 2 and the other in grade 5. May was supporting three primary school aged children, one in the first year of primary school, and the others in grades 3 and 4. To minimise potential confusion for the reader, illustrations used in this section are drawn just from two participants, Lois and Theresa.

#### Engagement

The facilitators actively participated in the mathematics tasks set by teachers with their children, supporting their children to interpret instructions, and to develop plans to complete set mathematics tasks. Throughout the interview, Lois constantly used phrases such as “we did” and “we were”, suggesting she worked with her son on much of his mathematics. She also described negotiating with her son what mathematics activities to do and when. Theresa similarly dedicated significant time to support her children’s learning, particularly for her youngest son. She said, “it took a lot of my time in terms of just scaffolding, just making him understand what the activity meant”.

#### Space

The facilitators exploited the affordances of the home learning space differently and more extensively than the monitors. In addition to using the flexibility of at-home learning to incentivise work, the facilitators moderated programs of learning in response to their child’s needs and their availability. Lois altered the teacher set learning programs in response to her work commitments, choosing easy activities for days when Lois was at work, whereas “something that, you know, needs a bit of guidance or enthusiasm he’ll do on the day that I’m at home”. Lois even dismissed some mathematics activities because of a lack of interest, a lack of resources, or a lack of perceived value. Similarly, Theresa adjusted the set mathematics program, responding to her son’s motivation levels. She said, “there are some days he wakes up and he doesn’t want to do stuff, you know… So, I’m not pushing much to be very honest”.

#### Capital

The facilitators accessed and activated some capital associated with mathematics or education, though all felt that this was not adequate. Lois described herself as a competent mathematician. She said, “I went into remote learning quite positive because I thought, you know, I’m reasonably clever. My son’s reasonably clever. We should be all over this. I proved myself very wrong”. Theresa said of herself that “math is not on the love list”; however, she was a qualified secondary science teacher in Bangkok for 10 years prior to moving to Australia, and so she had some understanding of education. She did not, however, feel she was familiar with techniques used for teaching mathematics, and this lack of capital had negative consequences. She said, “the teacher teaches in a certain way, we don’t know what way the teacher has picked up. And then, you know, you end up confusing them even more”.

The facilitators noted however some gains in capital through at-home learning, expressing the experience left them with a better understanding of their children’s mathematics abilities and some insight into mathematics education. Lois said, “I …hadn’t really ever done anything maths-wise with him. So, when we started home school the first time it was quite interesting”. She also said, “the positive for home schooling is that you see exactly where they’re at”. Theresa also valued the increased insight afforded by the shutdown, saying, “because of the closure, I’ve got a bit more control on what's going on. I can see what results are coming”.

The facilitators spoke about activating other capital to support at-home mathematics learning. Both were able to arrange the time to supervise at-home learning. Lois worked part-time at the hospital and was sometimes able to organise shifts to supervise at-home learning. Theresa was studying from home and her sons worked beside her. Both also spoke about accessing emotional capital throughout the shutdown to support at-home mathematics education. Lois said that her son “hated” mathematics during the school closure, and in response she said, “I try to be enthusiastic”. Similarly, Theresa empathised with her sons and adjusted programs to accommodate their emotional states. She said, “if they are not in the mood, it’s not because of the math … There are days when I don’t want to open my laptop”. The facilitators identified some deficits in capital. Lois spoke about the lack of availability of other family members to help supervise her son, and the lack of physical resources such as a printer to make hardcopies of resources sent home.

### Enhancers

Anna, Penelope, and Courtney were categorised as enhancers. Anna had two children, a daughter in grade 4 and a son in grade 6. Penelope had three primary school aged children, in grades 1, 4, and 6. Courtney had three children, two in primary school in grades 1 and 4, and one in preschool. To minimise potential confusion for the reader, illustrations used in this section are drawn just from two participants, Anna and Penelope.

#### Engagement

The enhancers actively critiqued the suitability of the mathematics tasks set to meet their children’s learning needs, and, when they believed necessary, created, or sourced alternative or additional learning experiences. Anna described the mathematics tasks set for her children as “very worksheet based” and felt this approach “wasn’t ideal”. She responded by modifying some elements of tasks that she felt were non-productive and supplementing the set work for her daughter with maths games and fluency tasks. She also developed and worked through concrete examples to support her son’s learning, saying, “he needed that modelling to be able to understand what to do before completing the worksheet or completing the online form”. Penelope would assess the set mathematics tasks and select activities to best support her children’s learning, focussing her efforts on her children’s relative academic weaknesses. She said, “I could look at the kids and think you have not got any lights on about this topic”. Penelope would respond to these issues by demonstrating how the mathematics could be applied. She said, “maths is always real… So, let’s make it a real thing, you know cutting up blocks of chocolate or pulling out string beads and doing all that sort of stuff”.

#### Space

As evidenced by the examples of engagement above, the enhancers exploited the opportunity created by the transfer of academic mathematics learning from the classroom space to the home space to adopt a lead role in their children’s mathematics learning. Furthermore, they saw themselves as central to the success of their children during at-home learning. Anna described her children as being happy with at-home mathematics education, saying, “they actually asked me, could I teach them all the time… I think they liked that one-on-one. And just being relaxed in their home environment”. Penelope said that her youngest son had told her that he liked having her teach him, and she noted that her efforts with her eldest son had resulted in 12 months growth in mathematics on his most recent report. She said, “I could provide all of their learning if I needed to”. Furthermore, the school closures revealed to Penelope her eldest son’s difficulties in mathematics. She sought professional help, resulting in a diagnosis of dyscalculia, dyslexia, and dysgraphia, and arranged a tutor to work with him throughout the closures.

#### Capital

The enhancers conveyed a sense of confidence that they possessed, and were able to activate capital in a way that appropriately supported their children’s mathematics education, though the capital they drew upon varied somewhat. The enhancers had significant capital associated with both mathematics and education. Anna, an education lecturer, noted she felt comfortable with the mathematics and the mathematics tasks her children were working on, as well as with the technology being used. Penelope was confident with mathematics, saying she was “a real maths and science lover at school”. Prior to the pandemic, she was engaged in her children’s schooling, saying, “I’m a parent that’s quite involved in our school” and she encouraged her children’s mathematics-related learning, as illustrated by the family’s monthly subscription to crates [boxes of resources], which are all that STEM sort of activities. Furthermore, she felt being a paediatric occupational therapist gave her “a really good skill set for me given that I realized a lot about my kids [due] to [the] enclosure [*sic*]”.

Beyond mathematics and education-related capital, the enhancers activated a good array of other capital to support at-home mathematics learning. The enhancers were able to find time to supervise at-home learning, with Penelope “juggling a pretty intense job”, and Anna working from home as a University Academic. Penelope activated financial capital, consulting professionals, and employing a tutor. Anna employed emotional capital to overcome the challenges of at-home learning which she described as “hard work and some days a nightmare”. Despite this, she said her children were “really enthusiastic” about at-home learning.

## Discussion and conclusion

This study reports on the findings from narrative interviews with eight parents who supported their child(ren)’s at-home mathematics learning during the 2020 school closures in Australian schools. This study identified three modes of parental engagement amongst participants: monitors (who saw their role primarily as ensuring mathematics tasks were completed without significantly involving themselves in the task), facilitators (who worked actively with their children on tasks to ensure their completion), and enhancers (who adjusted and supplemented mathematics tasks in an effort to best meet their children’s learning needs). It then analysed the engagement of parents in these categories through the lens of the Ecologies of Parental Engagement (EPE) revealing key differences in the way parents in these categories responded to the transition of academic mathematic learning to the home space, and also in the access and activation of capital to support their children’s learning.

There has been only limited research into parental engagement in mathematics education, and the typology of parental engagement in children’s mathematics learning emerging from this study appears new to mathematical research literature. Some of the behaviours of both monitors and facilitators have been researched previously. There is some research contrasting the impact of parental supervision of mathematics homework, and direct aid with homework (Patall et al., 2008; Rodríguez et al., 2017), behaviours typical of the monitors and facilitators, respectively. However, the available research does not identify behaviours typical of the enhancers. This may be because parental engagement is typically viewed in school-centred ways (Jay et al., 2018) where there is only a limited role for parents in academic mathematics learning spaces. The COVID disruption of schooling arguably created the conditions for the emergence of enhancer type engagement by translocating learning to the home and providing parents with the opportunity to take a key role in their child’s mathematics learning.

The first research question for this study was “How did parents engage in mathematics education during the COVID-19 school closures?” EPE posits that parental engagement is the outcome of parents interacting in a schooling space by accessing and activating available capital (Calabrese Barton et al., 2004). Analysis through this lens identified three characteristic patterns in the way parents responded to the COVID-disrupted learning space and their new role within it. The monitors used the home space to incentivise the completion of work, though posed no challenge to the teacher-set tasks or schedule. In contrast, the facilitators adjusted learning schedules in response to their children’s needs, while largely adhering to teacher-set mathematics tasks. The enhancers used the home space and their new role to most radical effect, both rescheduling tasks and modifying the learning tasks themselves. This use of home space for academic mathematics learning has no strong representation in the literature, given it was induced by the pandemic. Potentially informative literature might be research into parental experience of home schooling mathematics, but there is a paucity of relevant literature in this area (Reaburn, 2021).

The second research question for this study was “What factors impacted this engagement?” Parents in each engagement category had characteristic access to, and activation of, capital to support at-home mathematics learning, strengthening and extending on understandings from previous research in the field. The limited available evidence suggests that both parents’ knowledge of mathematics and their understanding of contemporary education shape their engagement in mathematics (e.g., Antolin Drešar & Lipovec, 2017; Jay et al., 2018). This study supports these findings, with monitors having limited knowledge of mathematics or education, facilitators having some understanding of either education or mathematics, and enhancers having some understanding of both. Furthermore, this study shows that the finding that socioeconomic background is related to the ease with which parents engage in schooling generally (Woodrow et al., 2016) certainly applies to parental engagement in mathematics education. Monitors tended to activate a less diverse array of capital—largely limited to their family and school provided resources—whereas the enhancers activated extensive capital, variously exploiting family, social, and professional networks and knowledge, and in some cases exercised significant financial capital, to support their children’s mathematics learning. Finally, this study highlighted the value of parental emotional capital in supporting their children’s mathematics learning. Supporting mathematics learning can be stressful for parents (Jay et al., 2018), and this was the case for all participants in this study. Emotional capital is used for emotional self-management (Reay, 2004). In this study, one monitor reported high frustration and conflict associated with supporting at-home mathematics learning, while the other did not engage with her child’s mathematics learning beyond checking task completion. The facilitators all described highly emotional experiences; however, they all reported that they managed these situations satisfactorily. The enhancers referred to their activation of emotional capital less directly, though it seemed no less important as they described the challenges of maintaining motivation and engagement in mathematics learning for their children. These findings suggest that emotional capital is a key resource for all parents engaging with their children’s mathematics learning—and is perhaps more crucial when other available capital is limited.

While this study did not attempt to measure the impact of the various modes of engagement on children’s learning, some insights were provided by parents’ comments about the success of their efforts in at-home mathematics education. The monitors expressed doubts that their efforts resulted in any mathematics learning gains for their children. These doubts are supported by research. Patall et al.’s (2008) synthesis of 14 studies found that parents who monitored mathematics homework had a negative impact on achievement. A study of 897 grade 5 and 6 students also found that direct supervision of mathematical tasks was not positively correlated with achievement (Rodríguez et al., 2017). The facilitators expressed concerns about their understanding of classroom mathematics education practices, a reasonable concern given that when parents offer active assistance in mathematics that is counter to their child’s experience in school, it can negatively impact learning (Lange & Meaney, 2011; Rodríguez et al., 2017). Again, there is a dearth of evidence about engagement typical of the enhancers; however, high parental support, expectations, interest in mathematics work, and confidence in children’s abilities have all been shown to contribute positively to children’s mathematics learning (Rodríguez et al., 2017; Vukovic et al., 2013), all of which were arguably communicated through the enhancer’s level and type of engagement with at-home mathematics learning. The enhancers’ positive perception of their impact on their child(ren)’s mathematics learning seems supported by the available evidence.

While this study offers a categorisation of some patterns of parental engagement in mathematics education, and descriptions of the use of the home space and the capital accessed and activated to maintain this engagement, further research is required to develop the typology. The typology has some support from the existing research literature; however, it is based on interviews with eight parents selected to be representative of the diversity in a Facebook group of 43 Australian parents. The members of this Facebook group were self-selected and all reported some level of active engagement in their children’s mathematics education. As such, they were unlikely to be a truly representative sample of the wider Australian population of parents negotiating the demands of at-home schooling. Research with a larger sample of parents, and parents from other nations, would be required to further develop the typology. In addition, the data collected in this study focussed on capital that participants both had access to, and activated. It provides no information about potentially useful sources of capital that participants had but did not activate. For example, some parents may possess expertise in mathematics or mathematics education, but may be unable to activate it due to work obligations or some other restriction. Research exploring how and why capital is accessed to support children’s mathematical learning would be useful. Furthermore, there is a group of parents, referred to by some of the parents in this study, who are not represented in this study—those who did not engage in their children’s mathematics learning during the school closures. This study provides no insight into how these parents negotiated the at-home learning space, or the capital they had access to or were able to activate. Future research should seek out these parents to better understand their experiences. Finally, the study examined parental engagement during the early school closures, during the initial stages of the pandemic in Australia. Since data for this study were collected, there have been further periods of at-home learning of varying characteristics and duration, providing additional opportunities to explore parental engagement.

While data informing this study were collected during the extraordinary conditions produced by the pandemic, the typology emerging through this study has implications for researchers of parental engagement, and for schools and educators wishing to facilitate parental engagement in mathematics. It challenges us to think of parental engagement as more than just a willingness to support school programs—parents can, and do, actively shape their children’s mathematics learning experiences. This study reveals that where parents have a role in mathematics learning, and appropriate capital, they can engage with high levels of agency. Schools need to work to ensure parents feel that they have a role in the academic mathematics learning space and that they have access to appropriate capital to successfully engage in this space. Schools need to share knowledge about contemporary mathematics education practices with parents—the facilitators commented on the positive impact of developing their understanding of these practices gained throughout at-home learning, whereas the enhancers demonstrated competency in these practices from the beginning of school closures. Finally, this study highlights the value of emotional capital when supporting children’s mathematics education, challenging schools to ensure they employ practices that do not exacerbate the stresses associated with engaging in mathematics learning for parents or children.

## Data Availability

Data transparency.

## Code availability

Software application of custom code.
